# The Effect of Conditioning Exercise on the Health Status and Pain in Patients with Rheumatoid Arthritis: A Randomized Controlled Clinical Trial

**Published:** 2014-07

**Authors:** Iran Jahanbin, Mahboobeh Hoseini Moghadam, Mohammad Ali Nazarinia, Fariba Ghodsbin, Zahra Bagheri, Ali Reza Ashraf

**Affiliations:** 1 Community Based Psychiatric Care Research Center, Department of Community Health Nursing, School of Nursing and Midwifery, Shiraz University of Medical Sciences, Shiraz, Iran;; 2 Department of Community Health Nursing, School of Nursing and Midwifery, Shiraz University of Medical Sciences, Shiraz, Iran;; 3 Department of Internal Medicine, School of Medicine, Shiraz University of Medical Sciences, Shiraz, Iran;; 4 Department of Epidemiology, School of Medicine, Shiraz University of Medical Sciences, Shiraz, Iran;; 5 Department of Medicine and Rehabilitation, School of Medicine, Shiraz University of Medical Sciences, Shiraz, Iran

**Keywords:** Rheumatoid Arthritis, Exercise, Pain, Health Status

## Abstract

**Background:** Rheumatoid Arthritis (RA) is a systemic and inflammatory disease of unknown etiology which is mostly characterized by inflammation of the synovial joints. Studies have proved that most people with RA avoid doing physical activities due to fear that it may worsen the pain or cause pressure on joints, resulting in decreased muscle strength and ultimately leading to disability of patients. We aimed to investigate the effects of conditioning exercises on the health status and pain in patients suffering from RA.

**Methods:** In this randomized controlled clinical trial, we enrolled 66 women with confirmed RA referred to the rheumatology clinic of Hafez hospital, Shiraz, southwest Iran during May-July 2013. Balanced block randomization method was used to randomize the participants into case and control groups (two groups of 33 each).Data were collected using visual analog scale (VAS), Arthritis Impact Measurement Scales 2 short form (AIMS2-SF), and demographic questionnaire. After obtaining written informed consent, the participants in the case group were asked to participate in conditioning exercise programs including aerobic, isometric, and isotonic exercises and received a training booklet explaining the exercises that they could do at home after the intervention.

**Results: **There was a statistically significant difference between the health status scores of the patients in the case groups before and after the intervention (P=0.001). The pain score also decreased significantly in the case group compared with the control group after the intervention (P=0.003).

**Conclusion: **We concluded that physical training programs, especially conditioning exercises, could improve the health status and reduce pain in patients with RA.

**Trial Registration Number: **IRCT201308187531N3

## Introduction

Rheumatoid Arthritis (RA) is a chronic disabling disease which is mostly manifested by inflammation of the synovial joints. This autoimmune disease results in progressive joint destruction and deformity leading to varying degrees of limitations in daily activities.^1^ The disease is one of the most common unknown diseases and the major cause of disability in adults. RA initially involves the small hand or feet joints and it spreads to the larger joints during its progression. While such disease may be transient, it usually becomes chronic and causes destruction of the joints sooner or later (in few months or year).^2^^,^^3^

According to several studies, its global prevalence rate is reported 0.5 -1% worldwide.^2^^,^^4^ However, its prevalence varies in different populations, for example in people with family history of RA; its rate is approximately 2-3%.^4^^,^^5^ Besides, the mortality rate of those affected is twice that of general population at the same age. The prevalence rate is significantly increasing in the recent years.^2^^,^^6^ Currently, more than 2 million people in the United States are suffering from RA while it is predicted that by 2020 the number of the patients increases to more than 18% due to the increased longevity of the population.^7^^,^^8^ Its prevalence and incidence increase with age. Furthermore, women are more likely to develop such disease than men as 70% of the patients suffering from the disease are women. Men are affected with more severe form of RA.

This disease, which mostly occurs in the fourth and fifth decades of life, can disrupt normal daily activities^2^^,^^9^ and may cause numerous physical complications. Chronic pain, fatigue, impaired mobility and limb deformities are the major complications induced by the disease.^10^ Its symptoms, such as stiffness of the joints and movement disorders, are progressive to the extent that a number of the patients lose their mobility and activity and are almost entirely paralyzed and crippled. Mobility problems may impose major damages and complications on both the patient and society, and may have negative effects on them physically, socially, psychologically and economically.^2^^,^^11^

It is essential to maintain physical performance in patients with RA so that they can perform self-care activities. Accordingly, regular exercise can decrease the pain, fatigue, and morning stiffness in patients and increase their self-confidence.^11^ Rheumatologic studies have shown that exercising could positively affect their quality of life by reducing muscle pain and stiffness.^12^ It can also reduce symptoms of anxiety and depression and the recurrence frequency of chronic pains.^13^

Studies have proved that most people with RA avoid doing physical activities due to fear that it may worsen the pain or cause pressure on joints.^14^^,^^15^ It can result in decreased muscle strength and aerobic capacity and ultimately lead to disability of patients.^15^ In patients with RA, the muscles around the joint may become weak if the joints cannot move strongly. Therefore, physical and exercise therapy is effective in pain reduction and improving range of motion around the joints by strengthening the muscles that surround the joint and reducing the pressure on them.

Performing physical exercises is beneficial in improving respiratory capacity as well as physical and functional strength in patients with RA.^11^^,^^16^ Physical activity,specially group exercising, can improve social interaction and increase the sense of well-being.^16^ The short-term effects of strengthening exercises on the patients with such disease have well been described in several studies; however, only a few studies have considered the long-term effects of exercising on the patients’ muscle strength and activity.^1^

Conditioning exercise is an activity which improves cardiovascular and muscle endurance as well as muscle strength including aerobic exercise, plyometrics, calisthenics, and exercises based on real-life motions. It increases the energy capacity of the muscle and is primarily involved in developing skills. Different types of conditioning exercises can be used depending on fitness goals as they could be adopted to any level of fitness (from beginners to experienced athletes).^17^

Besides, a study performed on 20 women with RA showed that isometric and isotonic strengthening exercises combined with 15 minutes of cycling not only do not worsen the disease but also improve physical strength and performance in such patients.^18^ The importance of exercising has been proven and clarified in the management and treatment of RA. Generally, physical exercise could improve the performance of the patients with such disease. Hence, all of the patients suffering from such disease should be encouraged to participate in some types of physical exercises as a part of their daily routine-activities. Therefore, we aimed to investigate the effects of conditioning exercises on the health status and pain in patients suffering from RA.

## Patients and Methods

**Figure 1:**
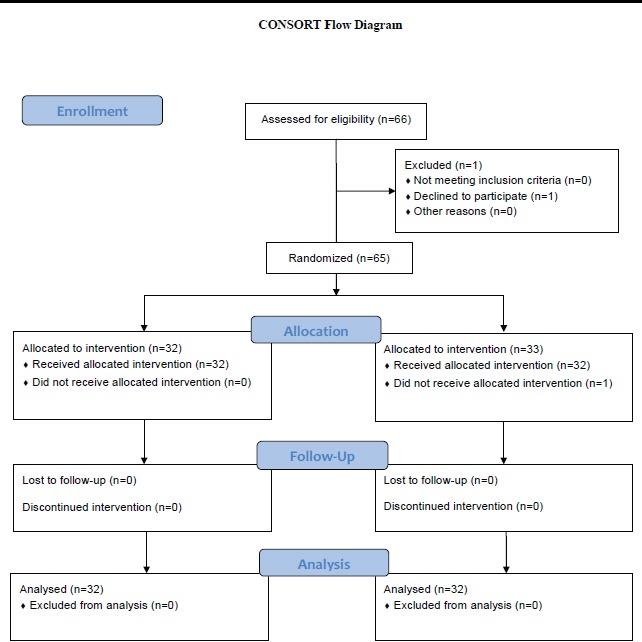
Consort dolow diagram of participants

This study was anopen randomized controlled clinical trial, approved by the Ethics Committee of Shiraz University of Medical Sciences. We enrolled 66 women with confirmed RA according to ACR 2010 (American College of Rheumatology) by a rheumatologist who referred to the rheumatology clinic of Hafez hospital, Shiraz, southwest Iran during May to July 2013. Of them, 2 were excluded from the study as they did not meet inclusion criteria or declined to continue participation. The protocol of the study participants has been shown in Figure 1. The patients were selected using a simple sampling (according to Inclusion criteria)method, and balanced block randomization method (using a table of random numbers) was used to randomize the final 64 participants, into case and control groups (two groups of 32 each).

The sample size was calculated as 32 in each group based on the data of a similar study conducted by Stefano Masiero (2007);^1^ also, using Power SSC statistical software (power: 80%, α: 0.05) and considering the following formula, the predicted loss samples were taken from 66 patients.

n=26(Z1-a/2+Z1-B)^2/(µ1-µ2)

The inclusion criteria were: confirmed diagnosis of RA, age of 18 or more, willingness to participate in the study, ability to perform the exercises twice a week, ability to understand and communicate in Persian language, residence in the city where the study was done, and lack of osteoarthritis or any other inflammatory articular diseases. Exclusion criteria were suffering from severe form of the disease, any changes in the treatment of articular therapy in the last 6 months or during the study, need to change the treatment, performing Joint surgery in the 6 months before the intervention, participation in a similar training programs, and absence of more than two sessions in training program. Besides, in case of need to change the treatment for the patients, they were excluded from the study; however, we had no such case.

After obtaining written informed consent, the researcher explained the aims and method of the research to the patients who were willing to participate in the study and asked them to complete the questionnaires. Data were collected using visual analog scale (VAS), Arthritis Impact Measurement Scales 2 short form (AIMS2-SF) and demographic questionnaire containing information about age, age of occurrence of the disease, duration of the disease, educational level, employment and marital status.

AIMS2-SF is a self-reported questionnaire with 12 scales to assess five components of health status in patients with arthritis: physical (12 items), symptom (3 items), affect (5 items), and social interaction (4 items), and role (2 items) components. The 26-item AIMS2-SF is a shorter version of the AIMS2 (originally designed by Meenanand colleagues, 1980) which was developed by Gullemin and colleagues.^19^ The items are answered with options ranging from “all days” to “no days” in the previous four weeks. All the items are measured on a five-point Likert scale scored from 1 to 5.The total scores range from 26 to 130. The reliability and validity of AIMS2-SF were assessed in some studies and were also estimated in Iran by Askary-Ashtiani and colleagues (2009) on 350 patients with RA and Cronbach’s alpha was calculated as 0.74-0.89.^20^

The pain VAS is an instrument in which pain intensity is rated on a four-point scale (scale one: 0-2; scale 2: 2-5; scale 3: 5-8 and scale 4: 8-10). The scale is anchored by “no pain” (first scale) and “worst imaginable pain” (fourth scale).

The case group then was divided into two groups of 16 participants each. They were asked to participate in conditioning exercise programs consisting of two 45-minutes sessions per week for 8 consecutive weeks and were trained by the researcher. Conditioning exercises included aerobic, isometric, and isotonic exercises. Moreover, the patients received a training complied with the routine program of the clinic.

Immediately after the intervention, the VAS and AIMS2 questionnaires were completed again by the participants of both case and control groups. Finally, the patients in the control group received the same booklet. The collected data were analyzed using SPSS software, version 18. The significance level was set at <0.05.

## Results

The age range of the patients was 23-63 years. Their mean±SD ages was 48.6±10.51 and 48.87±9.24 in the case and control groups, respectively. The mean±SD age of the occurrence was 39.00±10.23 and 40.28±7.80 years in the case and control group, respectively. According to independent t-test, there was no significant difference between the two groups with respect to their mean age (P=0.74) and mean age of occurrence (P=0.57).

The mean duration of the disease in the majority of the studied cases (64%) was more than 5 years and Chi-square test showed no significant difference between the two groups in this regard (P=0.20). 84.4% of the participants in the case group and 87.5% of those in the control groups were married (P=1.00). 50% of the patients in both groups did not have secondary education. Furthermore, 71.9% of the participants in the case group and 78.1% of those in the control group were housewives. 

Chi-square test showed no significant difference between the two groups as to the marital and occupational status as well as educational level. Both groups of the patients were homogeneous with respect to their demographic characteristics.

Table 1 shows that there was no statistically significant difference between the case and control groups in terms of pain score and health status before the intervention (P>0.05). However, a significant difference was observed between the two groups immediately after the intervention (P<0.001).

**Table 1:** Comparison of the mean±SD of pain score and health status before and after the intervention in the case and control groups

**Variables**		**Groups**	**mean** **±** **SD** **Before intervention**	**P value** **(Between Groups)**	**mean±SD** **After intervention**	**P value** **(Between Groups)**	**P value** **(Within Groups)**
Pain (Visual analog scale)		Case group	2.84±0.12	0.390	2.03±0.12	0.003	0.000
		Control Group	2.71±0.09		2.53±0.08		0.05
Health Status (AIMS2-SF)	Physical health	Case Group	6.41±1.84	0.370	8.08±1.24	0.001	0.000
		Control Group	6.80±1.61		6.76±1.70		0.603
	Pain and other symptoms	Case group	4.29±1.96	0.454	6.48±1.66	0.001	0.000
		Control Group	4.63±1.61		4.58±1.70		0.701
	Psychological health	Case Group	4.60±1.85	0.654	6.68±1.44	0.001	0.000
		Control Group	4.80±1.48		4.91±1.27		0.415
	Social interaction	Case Group	4.33±1.81	0.615	5.95±1.29	0.001	0.000
		Control Group	4.55±1.57		4.49±1.25		0.698
	Function	Case Group	5.54±2.04	0.894	7.94±1.69	0.001	0.000
		Control Group	5.47±1.84		5.78±1.73		0.161

## Discussion

We found no significant difference between the case and control groups with respect to the pain score before the intervention (P=0.39). Therefore, the participants were all in the same status regarding the amount of pain they experienced. However, the pain score decreased in both groups of the patients immediately after the intervention and such decrease was only statistically significant in the case group based on Wilcoxon test (P<0.001), demonstrating the effect of exercise training intervention on pain relief among the participants of this group. Therefore, exercise interventions, on one hand could be used as an appropriate solution in decreasing pain, and on the other hand, it could increase the ability of the patients to manage their own condition.

Our findings were consistent with those of the studies which confirm that participation in exercise programs is effective on pain reduction in patients suffering from RA. Moreover, Flint-Wagner and colleagues (2009) conducted a study on 24 patients with RA in United States to investigate the effect of a 16-week training program on strength, pain and function. They found out that training could decrease the pain and improve function in the patients.^21^ Similarly, according to the study conducted by Teybi-Sabet, performing appropriate physical exercises could desirably reduce the pain and slow the process of the disease.^22^

In a study conducted by Kalali Jouneghani and colleagues, the severity of pain in the participants in the case group decreased significantly compared with those in the control group.^23^ Mohammadzadeh and colleagues also revealed that exercising in water (hydrotherapy) could positively lead to pain relief in the patients with RA.^11^ Likewise, Cadmus et al. concluded that a 10-week aquatic exercise program could help to reduce joint pain and improve the quality of life in patients.^24^ Eyigor and colleagues also suggested that physical therapy techniques, such as physiotherapy and isometric exercises, could reduce pain in those with osteoarthritis.^25^

Furthermore, Masiero et al. compared the mean difference of the pain score between the two groups of participants after 8 months and found out that it was only statistically significant in the case group.^1^ Generally, management of such disease is established upon a treatment pyramid in which the pain relief is considered as its main base.^26^ Our findings showed no statistically significant difference between the two groups with respect to the mean score of all dimensions of health status before the intervention. However, the mean score increased immediately after the intervention and such increase was statistically significant in all dimensions of health (P<0.001).In the control group, the mean score increased in the dimensions of role and affect but decreased in the dimensions of physical health, symptoms and social interaction; however, such changes were not statistically significant in any of dimensions.

Our findings were similar to those of De Jong and colleagues (2003) since in their study, conducted on 306 patients with RA, a twice-a-week training program improved the functional ability and health status of the patients.^27^ Moreover, after conducting another study on 120 patients with knee osteoarthritis, the researchers reported improvements in the health status of the patients in the case group and no statistically significant changes in the control group.^28^

Besides, the findings of another study showed significant improvements in the patients’ strength, pain and function.^21^ Our findings were also inconsistence with some other studies, confirming the efficacy of training and exercise in the improvement of health status in patients suffering from RA.^1^^,^^29^^,^^30^ Similarly, Katz concluded that inability in performing daily activities is common among such patients.^31^

As mentioned before, physical complications of the disease are progressive to the extent that a number of such patients are almost entirely paralyzed and crippled. Therefore, rehabilitation programs should be used to help the patients to maintain their physical performance. They should also be taught how to cope with the disease and its consequences.^2^^,^^11^ Previous studies have shown that physical exercise trainings and interventions could improve the health status of the patients with chronic diseases.^32^

Nurses and their abilities in training the patients have an old history as Nolan believes that nurses can do interventions in the field of pain relief, nutrition and mobility, self-care, creating positive images and increasing self-confidence in the patients.^33^ Besides, community health nurses can help their patients to maintain flexibility, muscle strength, and bone mass by counseling about nutritional practices and encouraging adoption with exercising which can improve the muscles strength.^16^

## Conclusion

It can be concluded that physical training programs,especially conditioning exercises including aerobic, isometric, and isotonic exercises,could improve the health status and reduce pain in the patients with RA. Physical exercising is an appropriate intervention on the patients since it could not only maintain the patients’ physical health but also positively affect their psychological and social dimensions. However, more research is required to examine the stability of the efficacy of training programs on the health status and pain relief in the patients.
